# Pathogen spreading during the COVID‐19 pandemic: Understanding a global phenomenon

**DOI:** 10.1002/puh2.71

**Published:** 2023-03-15

**Authors:** Peter B. Crabb

**Affiliations:** ^1^ Department of Psychology Pennsylvania State University Hazleton Pennsylvania USA

**Keywords:** assault, coughing, COVID‐19, news items, pandemic, pathogen spread, respiratory fluids, spitting

## Abstract

This study examined news media reports published during the early months of the COVID‐19 pandemic that described people spitting, coughing, and otherwise spreading respiratory fluids to other people and objects. A search of a news archive yielded more than 800 news articles published during March, April, and May, 2020, from which *N* = 325 cases of intentional pathogen spreading were identified. This was during the early part of the pandemic when the world was still trying to reach an understanding of how to deal with the pandemic. Collected news articles showed that cases of intentional pathogen spreading were reported to have occurred in 14 countries on 5 continents on most days (78.3%) of the 3‐month period considered. In 43% of cases, perpetrators claimed to be infected with the SARS‐CoV‐2 virus. Frontline key workers, passersby, and retail workers were the most frequent targets. The findings suggest that more needs to be learned about intentional pathogen spreading behavior, with the goals of reducing its occurrence in future pandemics and protecting vulnerable targets.

## INTRODUCTION

In the early months of the COVID‐19 pandemic in 2020, scattered news stories appeared reporting graphic acts of people spitting and coughing on others and on food in grocery stores, sometimes while claiming to be infected with the SARS‐CoV‐2 virus [1]. The severe distress experienced by people during the COVID‐19 pandemic [2] may have increased the potential for interpersonal aggression throughout affected populations [3]. With already anxious and stressed populations as a result of lockdowns, job losses, and inability to do their normal routines, mental health and social relationships continued to be challenged as manifested by aggressive behaviors and interpersonal conflicts. The risk to public health of intentional acts of spreading pathogens during a disease outbreak such as the COVID‐19 pandemic would likely be higher rates of infection and, possibly, deaths. In some countries, such acts were considered not only offensive but a criminal offense. To better understand the disturbing phenomenon of intentional pathogen spreading, a study was initiated to find and assess evidence that people attempted to spread respiratory fluids and the germs they may have carried during the COVID‐19 pandemic.

## INTENTIONAL PATHOGEN SPREADING

The author searched a news archive (NewsBank, http://newsbank.com) that contained “current and archived news content from more than 6900 sources spanning 200+ countries and territories.” A sample of *N* = 92 days of new reported cases of pathogen‐spreading was required to achieve a power level of 0.80 at *p* < 0.05. English‐language news reports were retrieved for this study using the search terms “spit,” “spitting,” “cough,” “coughing,” “sneeze,” “sneezing,” and “coronavirus.” As these behaviors showed significance in the possible spread of this infectious disease on top of the sensitivity of a fast‐spreading pandemic causing massive hospitalizations and deaths, newspapers highlighted these issues and provided some weight on its news reporting.

The period considered for this study was March 1 through May 31, 2020, during which the World Health Organization declared the COVID‐19 outbreak to be a pandemic, many countries declared national emergencies, travel restrictions were enacted, and schools and businesses were closed [4]. This was the period when many public health programs were strictly enforced, including lockdowns and quarantines. This time frame was characterized by the chaotic sourcing for personal protective equipment for health personnel, medicines for confined COVID‐19 patients, lonely deaths of patients in hospitals, and the rapid search for vaccines. This was also a period when governments were scrambling for solutions to many of the issues brought about by the pandemic.

The author assigned numerical codes and recorded information contained in the text of the retrieved news articles, including publication date, country, setting in which the attack occurred, pathogen spreading act (spitting, coughing, sneezing, and others), perpetrator's age and gender, perpetrator's claim to have an infectious disease, and the target of the attack. The coding procedure reached excellent levels of code–recode reliability (*r*s = 1.00). In the event of multiple news articles about a single case, the chronologically earliest article was included in the sample, and subsequent articles were excluded, which ensured that only new cases were counted on each of the 92 days of the observation period.

**FIGURE 1:**
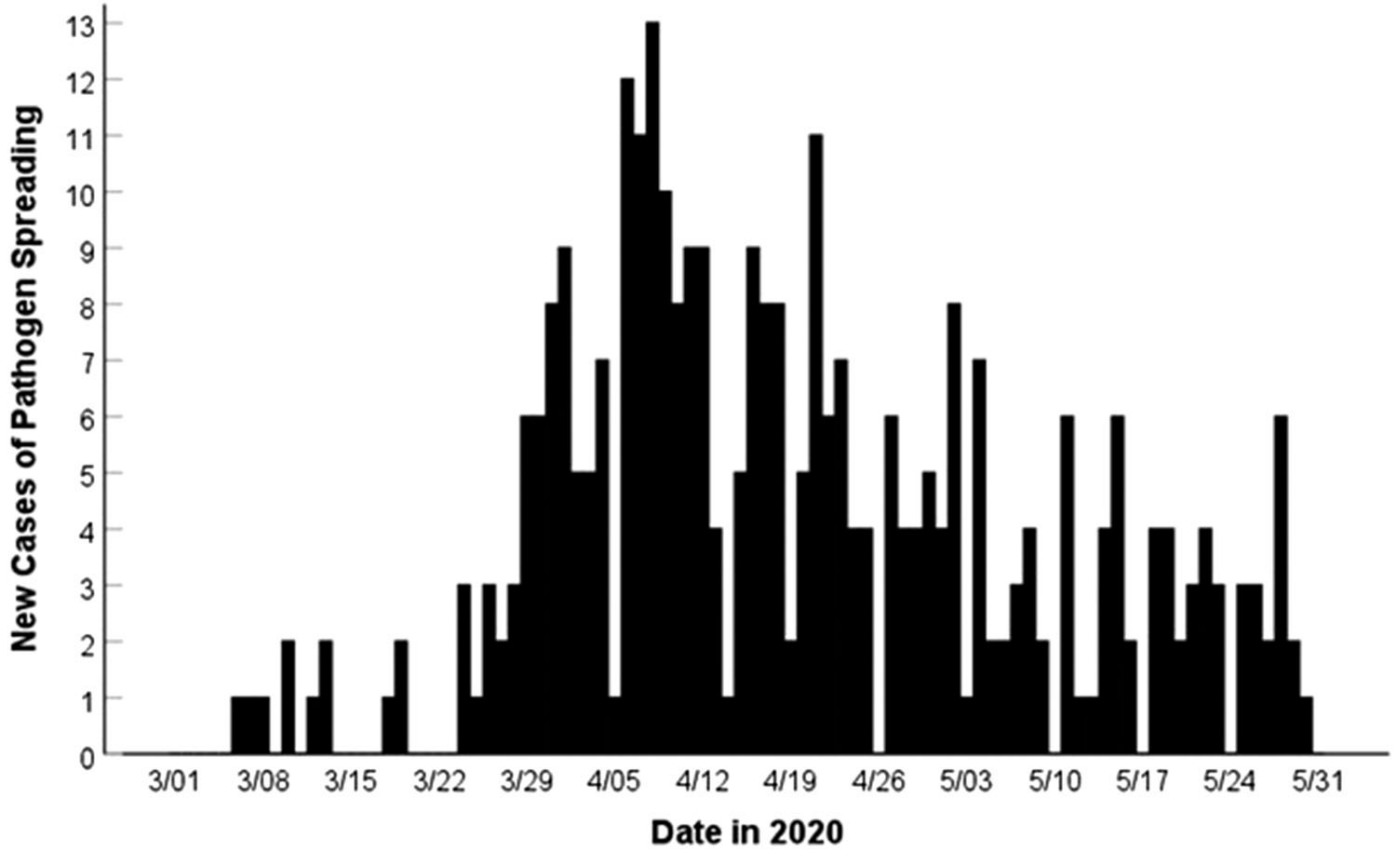
Daily frequencies of new cases of pathogen spreading reported in 14 countries during the spring, 2020, period of the COVID‐19 pandemic (*N* = 325).

The search in the NewsBank.com database yielded more than 800 articles published during the 92‐day period. After eliminating duplicate and nonrelevant articles, a final sample of *N* = 325 pathogen spreading cases was obtained and used in the analysis and counts. The daily frequencies of reported pathogen spreading cases are displayed in Figure 1.

New pathogen spreading cases were reported on most days (78.3%, *N* = 72) of the 92‐day observation period (*Χ* ^2^(1, *N* = 92) = 29.39, *p* < 0.001, *ω* = +0.56, 95% CI [69.83, 86.69]). The number of new cases per day ranged from 0 to 13, with a median of three cases per day.

As Table 1 shows, pathogen spreading cases were reported in 14 countries on 5 continents showing the universality of the phenomenon.

**TABLE 1:** Percentage of pathogen spreading cases by country (*N* = 325).

United Kingdom	40
United States	29
Australia	16
Canada	4
Ireland	3
India	3
New Zealand	3
Thailand	<1
Belgium	<1
Cyprus	<1
Israel	<1
Malaysia	<1
Portugal	<1
Saudi Arabia	<1

Spitting (87%) was the most common pathogen spreading act, followed by coughing (9.2%), smearing (1.5%), sneezing (0.6%), sharing food or beverage (0.3%), biting (0.3%), and spraying with a spray bottle (0.3%). Pathogen spreading acts occurred in 35 settings. The majority (74%) of cases occurred in homes (14.2%), public transit (10.4%), retail stores (10.4%), roadways (8.6%), grocery stores (8.3%), hospitals (6.8%), police stations (4.9%), police vehicles (4.3%), restaurants and bars, and public spaces.

Males were perpetrators of pathogen spreading in 70% of cases, with females implicated in 30% of cases. Ages of perpetrators ranged from 13 to 71‐year old (*Median* = 33 years). In 43% of cases, perpetrators claimed to be infected with the SARS‐CoV‐2 virus.

Twenty types of targets of pathogen spreading attacks were reported, most of which were frontline key workers (62.4%: law enforcement, EMS staff, medical staff), passersby (12.3%), retail store staff (9.5%), objects (6.4%: bank machine, car door handles, food in stores, doorknobs, elevator buttons, delivery packages, park benches, and train handgrips), and public transit workers (5.5%). Many of these targets are persons of authority who perform salient societal functions particularly during the pandemic.

A few quotes from the retrieved news reports illustrate the pathogen spreading acts:

“authorities arrested a man who removed his face mask, licked his fingers and then wiped them on a pole in a train despite the spread of coronavirus [5].”

“A 33‐year‐old woman claiming to be infected with COIVID‐19 allegedly spit in a nurse's face early Monday [6].”

“a suspect [police] arrested reportedly told them he had coronavirus and proceeded to cough in their vicinity [7].”

The findings support the conclusion that people did in fact intentionally attempt to spread pathogens during the early months of the COVID‐19 pandemic though these would need further unpacking. The risk to public health posed by this type of behavior is unknown at this time, but it is surmised that such acts can pose health threats to communities anywhere in the world.

This behavior may not be historically unique to the COVID‐19 outbreak. During the early years of the HIV/AIDS epidemic in the 1980s, cases were reported of people intentionally exposing others to HIV through sexual contact, biting, and spitting [8]. Future research into the occurrence of pathogen spreading behavior during previous pandemics (e.g., black death and Spanish influenza) might find evidence that the behavior is a predictable side effect of disease outbreaks.

## CONCLUSION

The information contained in these news stories tends to be limited. Inferences about perpetrators’ mindsets cannot be made until more data are gathered from richer sources such as interviews with detained perpetrators. The data reported here do suggest that, in many cases, interactional contexts with high conflict potential may have triggered spitting, and others, as defensive actions against perceived threats (e.g., police, EMTs, and ED staff). However, that would not explain why, in a small percentage of cases, some perpetrators chose to create “booby traps” by applying bodily fluids to objects that other people would touch or even eat. These cases deserve further scrutiny and suggest that it may be possible to distinguish between “defensive pathogen spreading,” as when a perpetrator spits at people they perceive to be threats, and “offensive pathogen spreading,” in which the perpetrator engages in clandestine spitting, and others, as retribution or a prank, for example. Additional research should examine whether (and how) the motives for these two types of acts may be distinct.

Although these data cannot shed light on why perpetrators chose to weaponize bodily fluids, the fact that spitting requires no planning, little effort, and no skill, and that it can have a substantial impact on targets, suggests that it can be a convenient, albeit antisocial, behavioral response to challenging situations. Weaponizing COVID‐19 also provides insights into a form a power relation between the attacker and the attacked. Future research will be required to more fully understand the contextual and cognitive factors that trigger this behavior, as well as to develop effective educational programs and prevention protocols to mitigate threats to public health [9]. Different research methods can be employed to understand the phenomenon, the knowledge of which will provide good scientific basis for policy and practice and inform actions in future epidemics and pandemics.

## AUTHOR CONTRIBUTIONS

All phases of this research were conducted by the author.

## CONFLICT OF INTEREST STATEMENT

The authors declare no conflict of interests.

## ETHICS STATEMENT

This research was approved by the Office for Research Protections, Pennsylvania State University.

## Data Availability

The data set and coding key are available online at https://doi.org/10.26208/tbfn‐px18.
